# 207. Burden of Post-Transplant Neutropenia and Leukopenia among Kidney Transplant Recipients: A Multi-Institutional Real-World Observational Study

**DOI:** 10.1093/ofid/ofac492.284

**Published:** 2022-12-15

**Authors:** Amit D Raval, Vladimir Turzhitsky, Elnara Fazio-Eynullayeva, Harry Jin, Sanjay Merchant

**Affiliations:** Merck & Co., Inc., Rahway, New Jersey; Merck & Co., Inc., Rahway, New Jersey; TriNetX, LLC, Cambridge, Massachusetts; TriNetX, San Francisco, California; Merck & Co., Inc., Rahway, New Jersey

## Abstract

**Background:**

Kidney transplant recipients (KTRs) are commonly prescribed valganciclovir/ganciclovir (V/G) prophylactically to prevent cytomegalovirus (CMV) infection; however, prolonged exposure to these medications is associated with an increased risk of post-transplant neutropenia (PTN) and leukopenia (PTL). Real-world evidence characterizing the incidence of PTN and PTL and their associated consequences on a national level are limited.

**Methods:**

This retrospective cohort study utilized the TriNetX Dataworks – USA Network, a global federated network of de-identified electronic health record (EHR) data for 82.5 million patients across 49 US healthcare organizations. Adult KTRs who were treated with V/G between January 1, 2012 and September 30, 2020, were included in this analysis. PTN was defined as absolute neutrophil count< 1500/μL and PTL was defined as white blood cell count< 3,500/μL. We estimated the proportion of PTN and PTL among KTRs and examined differences in granulocyte colony-stimulating factor (G-CSF) use and clinical outcomes between KTRs with and without PTN or PTL in the 1-year following KT.

**Results:**

Overall, 8,791 patients included in the analyses had a mean age of 52.8 years, 40.7% were females, 41.6% White and 32.6% Black. 3,383 patients (38.5%) developed PTN and 6,127 patients (69.7%) developed PTL based on the laboratory definitions. The incidence rates of PTN and PTL were 15.5/100 person-days and 53.4/100 person-days, respectively. There were no significant differences between comparison groups with respect to demographic characteristics at baseline. KTRs with PTN had higher G-CSF use compared to those without PTNs (38.9% vs.3.61%). Similarly, those with PTLs had higher G-CSF use compared to those without (22.8% vs. 4.2%). Both PTN and PTL were also associated with increased risk of CMV infection, graft rejection, and loss (Table 1, all p< 0.001).

**Figure:**
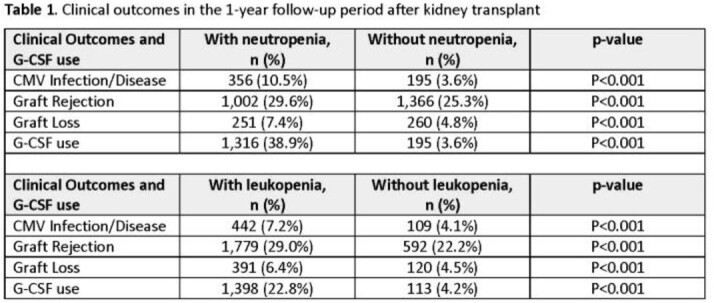

**Conclusion:**

The results of this large EHR study demonstrate that a sizeable proportion of V/G treated KTRs develop PTN and/or PTL, both of which are associated with increased medication use and suboptimal health outcomes. These findings underscore the importance of taking steps to mitigate the risk of neutropenia and leukopenia where possible to help improve outcomes in KTRs.

**Disclosures:**

**Amit D. Raval, PhD**, Merck and Co., Inc.: Employee of Merck|Merck and Co., Inc.: Stocks/Bonds **Vladimir Turzhitsky, PhD**, Merck & Co., Inc.,: Employee|Merck & Co., Inc.,: Stocks/Bonds **Sanjay Merchant, PhD**, Merck & Co., Inc.: Stocks/Bonds.

